# Antimicrobial Polymeric Structures Assembled on Surfaces

**DOI:** 10.3390/polym13101552

**Published:** 2021-05-12

**Authors:** Iulia Babutan, Alexandra-Delia Lucaci, Ioan Botiz

**Affiliations:** 1 Interdisciplinary Research Institute on Bio-Nano-Sciences, Babeș-Bolyai University, 42 Treboniu Laurian Str., 400271 Cluj-Napoca, Romania; iulia.babutan@ubbcluj.ro; 2 Faculty of Physics, Babeș-Bolyai University, 1 M. Kogălniceanu Str., 400084 Cluj-Napoca, Romania; 3 George Emil Palade University of Medicine, Pharmacy, Science, and Technology of Târgu Mureș, 38 Gheorghe Marinescu Str., 540142 Târgu Mureș, Romania; lucaci.alexandra-delia@stud16.umftgm.ro

**Keywords:** synthetic antimicrobial polymers, assembled nanostructures, surfaces and coatings, antimicrobial properties

## Abstract

Pathogenic microbes are the main cause of various undesired infections in living organisms, including humans. Most of these infections are favored in hospital environments where humans are being treated with antibiotics and where some microbes succeed in developing resistance to such drugs. As a consequence, our society is currently researching for alternative, yet more efficient antimicrobial solutions. Certain natural and synthetic polymers are versatile materials that have already proved themselves to be highly suitable for the development of the next-generation of antimicrobial systems that can efficiently prevent and kill microbes in various environments. Here, we discuss the latest developments of polymeric structures, exhibiting (reinforced) antimicrobial attributes that can be assembled on surfaces and coatings either from synthetic polymers displaying antiadhesive and/or antimicrobial properties or from blends and nanocomposites based on such polymers.

## 1. Introduction

The risk of microbial infection associated with multidrug-resistant microbes, i.e., certain microbes that have developed resistance to at least one drug of three different antimicrobial drug categories, has become an increasingly important problem for human health [1,2,3,4]. An efficient way to prevent and control possible microbial infections is the administration of antibiotics [5]. Nonetheless, excessive consumption of antibiotics leads, for instance, to developing new strains of antibiotic-resistant bacteria [6]. This happens inclusively in hospitals where patients with compromised immunity are treated for chronic diseases. Moreover, in recent years, the development of new types of antibiotics has been hindered by significant scientific challenges, regulatory uncertainties and industrial difficulties [7]. As a result, millions of people are infected yearly with multidrug-resistant bacteria [8], and the number of deaths associated with such infections is significant [9]. Furthermore, considerable developments of biomaterials led to the appearance of vital medical devices, such as catheters, joint implants or contact lenses, just to name a few. Unfortunately, while the surfaces of these devices are constantly exposed to microbial adhesion [10], treating the eventual infections requires significant efforts [11] and therapeutic targets [12].

Antimicrobial polymers are (biocidal) materials that can prevent and suppress the growth of various undesired microorganisms, including bacteria. Moreover, they can combat the bacterial resistance to antibiotics because, unlike conventional antibiotics, polymers exhibit antimicrobial mechanisms that cannot be outwitted by pathogens [13]. Furthermore, antimicrobial polymers can be easily adapted to applications, such as coatings and used to sterilize various surfaces, inclusively those of medical instruments. Thus, such polymers could become a good alternative to antibiotics and disinfectants and, why not, could eventually replace them in the future. This could be possible, especially because polymers come with important advantages, as they can adopt more or less complex chemical structures [14,15] that can favor their assembly and crystallization processes. These processes actually dictate the final properties of polymers in bulk, solutions, or thin-films [16,17,18,19]. Due to the potentiality to precisely control other processing parameters, such as melting, crystallization or glass-transition temperature, a polymer can display a highly tunable molecular ordering on multiple-length scales, ranging from nanometers to macroscopic dimensions that can generate a diverse landscape of nanostructures [16,20,21,22]. Further expansion of this landscape on the molecular, microscopic and macroscopic scales can be induced by favoring physical and chemical interactions of specific chain segments with their neighbors [15,16,23] or by degrading the phase purity through the addition of other (polymeric) components [24,25,26,27].

Antimicrobial polymeric systems include biopolymers and synthetic polymers (hereafter simply “polymers”). While biopolymers, such as polypeptides, polysaccharides or polynucleotides, are natural chains produced by the cells of various living organisms, polymers are human-made from precursors, such as petroleum derivatives or even biological components like peptides [28], lysine or arginine [29]. Antimicrobial polymers are designed to imitate the antimicrobial structures produced by the immune systems of various living organisms to kill microbes and can contain in their backbone or their side-chains various moieties with biocidal properties.

In this work, we emphasize the antimicrobial properties of recent structures (self-)assembled from polymers on various surfaces, thin films and coatings. We highlight these antimicrobial structures and the corresponding antimicrobial mechanisms developed not only in pure polymeric materials but also in polymer-based nanocomposites and polymer–polymer or polymer–biopolymer blends, respectively.

## 2. Main Antimicrobial Mechanisms Associated with Polymeric Structures

To depict how significant is the role of antimicrobial polymers in the war against microbes, we start our review with a brief classification of the polymer-based antimicrobial mechanisms (Table 1). These mechanisms of action are mainly associated to polymeric structures loaded with drugs or formed in hydrogels, or bound to surfaces.

### 2.1. Drug-Loaded Polymers and the Associated Antimicrobial Mechanisms

**Figure 1:**
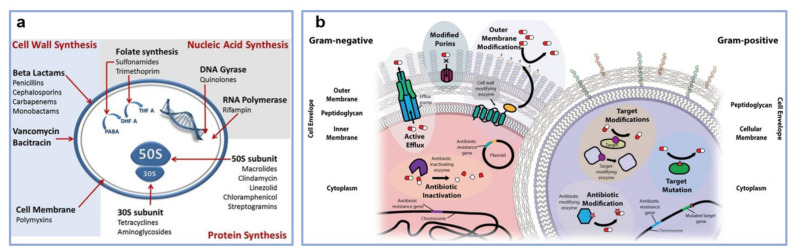
(**a**) Schematics depict the mechanism of action of antibiotics. (**b**) Illustration of mechanisms of antibiotic resistance corresponding to Gram-negative and Gram-positive bacteria. In comparison to Gram-positive bacteria, Gram-negative bacteria display an additional outer lipid membrane layer. This layer has the role of obstructing the entry of drugs into the bacterial cell, leading to antibiotic resistance. Several components contribute to this process: efflux pumps, porins with modified or reduced expression and cell wall modifying enzymes. On the other hand, Gram-positive bacteria present a thicker peptidoglycan layer whose structure can be altered to decrease antibiotic uptake. Enzymes encoded in Gram-positive and Gram-negative bacterial genetic material can inactivate or modify antibiotics. Furthermore, certain enzymes can protect the target of antibiotics by modifying their structure and/or number. Adapted with permission from ref. [47] (**a**) and ref. [48] (**b**).

Polymeric nanoparticles (NPs) are self-assembled (hierarchical) structures that can be rapidly adapted to achieve controlled and targeted drug load and release at the site of microbial infections by regulating the characteristics of polymers and the surface chemistry of resulting structures. Polymeric structures loaded with biocides can be concentrated preferentially at the infected site and can act as a depot that provides a continuous supply of encapsulated therapeutics over days or even weeks (see the mechanism of action of antibiotics schematically depicted in Figure 1a). A significant advantage of a drug delivery system based on polymeric NPs is that it can protect therapeutic agents against enzymatic degradation, while the dose required for the drug to be therapeutically efficient is significantly lower. Consequently, its systemic toxicity is reduced. Other advantages of polymeric NPs include the possibility to augment the bacterial sensitivity to drugs by overcoming two resistance mechanisms, namely increased efflux and decreased antibiotic uptake (Figure 1b), and to achieve curative effects more easily by packaging multiple drugs within the same NP, or by combining several types of NPs. This strategy enhances the antibacterial effects and helps preventing bacterial resistance [41].

Some polymeric self-assembled NPs have their antibacterial capacity from their multimodal mechanism of action: outer membrane destabilization, inner membrane perturbation and unregulated ionic movements, leading to apoptotic-like death followed by bacterial cell lysis [42,43]. Star polymer NPs [42] can encapsulate guest drug “cargos” with fewer undesired bursts [30] and can maintain solubility and viscosity similar to that of low molecular weight linear or branched polymers [44]. Although some star polymer NPs may be toxic and can present off-target side-effects against probiotic bacteria [42], the unwanted effects can be eliminated by adding sugar-based moieties on star polymers to target the receptors expressed on macrophages and to render polymers antimicrobial [45]. Similarly, glucosamine-functionalized star polymers can be employed to penetrate the peptidoglycan layer of bacterial cells more easily [46].

Polymeric micelles are core-shell NPs, typically formed through the self-assembly of amphiphilic block copolymers, in which the core can accommodate hydrophobic drugs, while the shell makes the micelle water-soluble and promotes delivering poorly soluble agents. They can also act as antimicrobial polymers by themselves without incorporating other biocidal agents [49]. Early biodegradable antimicrobial polymeric micelles, self-assembled from amphiphilic polycarbonates (PCs), exerted biocidal action by a cell wall and membrane disruption, which led to bacterial lysis [50]. The most important advantages presented by this system are the low hemolytic activity and low toxicity observed on mice. However, these micelles are ineffective against Gram-negative bacteria. To overcome this major drawback, a random copolymer structure can be used instead of a block copolymer, making the hydrophobic moieties more accessible, therefore, increasing the likelihood of membrane insertion and disruption [51]. Furthermore, multifunctional micelles, capable of both detection and inhibition of bacteria [52], as well as NPs exhibiting inherent antimicrobial activity and drug-loading capability, can be produced [53]. Other types of micelles include antimicrobial rod-shaped micelles that can be used efficiently against *C. albicans*, fungal species that are generally more difficult to kill due to their multilayered, thicker, and less negatively charged cell walls [31,32]. Although there were numerous attempts to create effective, biocompatible, and nontoxic polymeric micelles, the role of self-assembly on biological activity was not completely defined, with multiple studies declining the positive contributory role of micellization [54,55,56]. Moreover, it was postulated that the polymer chains might be more active as free molecules in solution. In contrast, the supramolecular structures could reduce the polymer chain mobility [54], hinder the exposure of cationic groups, which are essential for electrostatic interaction with bacterial cell walls [42] or shield the hydrophobic components required for the interaction with bacterial membrane within the core [57].

Polymeric vesicles (polymersomes) are other spherical polymeric capsules with a hollow inner compartment confined by a bilayered membrane composed of amphiphilic block copolymers that can be loaded with both hydrophobic and hydrophilic drugs. A distinctive characteristic of polymersomes is that they offer three different regions available for functionalization, more precisely the inner hydrophobic cavity, the polymer shell, and the periphery [58]. Stronger polymer–bacteria affinity exists when the polymers are self-assembled as vesicles, rather than in cases where polymer chains remained unassembled, probably due to increased local concentration of positive charges [33]. This is the case of perforated high-genus polymer vesicles that can encapsulate doxorubicin and manifest an acid-accelerated drug release profile that can be used for targeted therapeutic delivery when simultaneous antibacterial and anti-cancer medication is needed [59].

Dendrimers, i.e., highly branched symmetrical 3D macromolecules with core–shell architecture and nanometer-scale dimensions, consist of a (hydrophobic) core, layers of branched repeating units and an outer (hydrophilic) layer of functional end groups [60,61]. Although the diffusion of dendrimers is limited, initial adsorption and binding to cell membranes are stronger than for linear polymers [34]. In this case, biocidal hydrophobic and hydrophilic agents can be loaded inside the dendritic structure by noncovalent encapsulation or can be attached to surface moieties by covalent binding [61,62] followed by a targeted release that can increase the duration of action of the antimicrobials [63,64,65]. This is, inclusively, the case of cationic dendrimers that can eventually be loaded with antiproliferative constituents and can be employed as novel antibacterial agents against both Gram-positive and Gram-negative bacteria [66,67,68]. Usually, the bacteria-killing process is following these steps: adsorption onto negatively charged bacterial cell wall, diffusion through the cell wall, binding to the cell membrane, disruption and disintegration of the membrane followed by the release of electrolytes and nucleic materials from the cell, leading to death [62].

### 2.2. Antimicrobial Mechanisms Specific to Polymeric Hydrogels

Hydrogels are hydrophilic, crosslinked polymer networks with a unique 3D structure, capable of absorbing more than 20% of their weight of water while maintaining their structure and ability to control the release of therapeutics. They can be manufactured of polymers that are capable of converting to gels in the presence of different stimuli, such as temperature, pH, UV irradiation, etc. Hydrogels can be manufactured in a manner that confers mechanical features and structures similar to natural tissues. Because of their high biocompatibility, mucoadhesion and ability to respond to microenvironmental changes, hydrogels are attractive drug-delivery systems, especially for local antimicrobial applications. They can be successfully used as coatings for catheters, wound dressings, contact lenses, etc. The use of hydrogels loaded with antimicrobial therapeutics promotes delivering an adequate dose directly to the infected site and offers a stable and prolonged release, without undesired bursts, thus hindering the development of new drug-resistant strains. They offer a high surface-to-volume ratio and, most importantly, their hydrophilic nature provides an excellent solubilizing environment for numerous antibiotics like ciprofloxacin, amoxicillin, gentamicin [35].

Another advantage of antimicrobial hydrogels consists in the possibility to either construct hydrogel networks with inherent antibacterial activity by utilizing polymer molecules that exert antimicrobial properties by themselves [69,70] or to further employ at least two components exhibiting antimicrobial properties to generate hydrogels with synergistic effects that can further enhance the overall antimicrobial efficiency [71,72,73]. For instance, hydrogels are often loaded with inorganic NPs with great potential in biomedical applications [35,36]. Silver (Ag) is a popular antimicrobial agent that expresses biocidal properties against a wide range of bacteria, fungi and viruses due to its multiple mechanisms of action (including the release of Ag^+^, the intrinsic antibacterial properties of Ag-based on its penetration into the bacterial membrane and cell walls, the antibacterial effects caused by reactive oxygen species/ROS generated by Ag^+^ that can induce oxidative stress in bacterial cells) [74]. For example, exposure of microbes to Ag^+^ activates the interaction with proteins of the microbial cell wall and membrane, leading to the impairment of cellular transport systems, electrolytes imbalance, membrane perforations, loss of cytoplasmic organelles, alteration of bacterial cell division and finally, to cell death [75]. Thus, loading polymeric hydrogel matrices with Ag NPs leads to highly antimicrobial systems capable to efficiently neutralize Gram-positive and Gram-negative bacteria over longer periods [35,36]. Gold (Au) NPs also possess antibacterial properties and can be embedded within hydrogel networks. Although Au NPs are not as efficient as Ag NPs, they can cause bacterial membrane disruption, inducing cell death, have a large antibacterial spectrum, including methicillin-resistant *S. aureus* and *P. aeruginosa* and are nontoxic to osteoblastic cells [35,36]. Furthermore, ZnO shows antibacterial activity and low toxicity against mammalian cells. Its mechanism of action is based on damaging the lipids and proteins of the bacterial cell membrane and ROS generation. Significant peculiarities of ZnO NPs include their activity against high-temperature-resistant and high-pressure resistant bacterial spores and beneficial influence on bone regeneration [35,36]. Beside hydrogels, Ag, Au, ZnO and other types of inorganic NPs can also be incorporated in antimicrobial polymer composites in order to boost the antimicrobial properties of the latter, as we will see in Section 5.

### 2.3. Surface-Bounded Polymers and the Corresponding Antimicrobial Mechanisms

**Figure 2:**
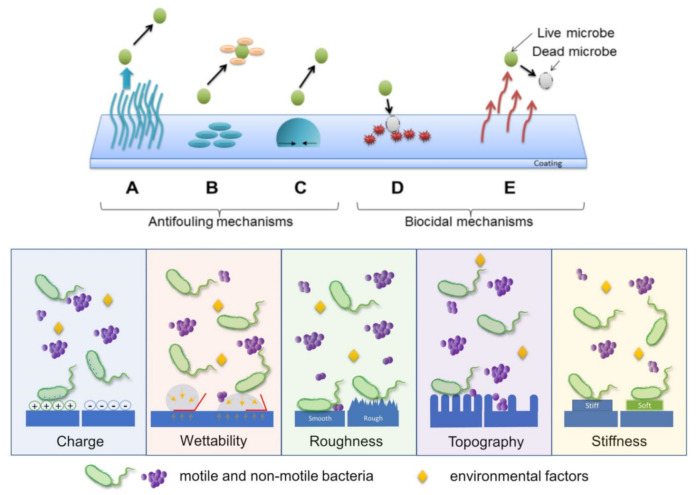
Examples of antimicrobial mechanisms of polymers: (**A**) steric repulsion, (**B**) electrostatic repulsion, (**C**) low surface energy (i.e., surfaces exhibiting high contact angle), (**D**) biocide releasing, (**E**) contact-killing of microbes (top). Schematic illustration depicting various surface parameters that exert significant influence on bacterial adhesion (bottom). Reprinted with permission from ref. [37] (top) and ref. [38] (bottom).

As it is schematically depicted in Figure 2 (top), there are two ways to keep various surfaces microbial-free: to prevent the attachment of microbes, including bacteria, on surfaces before any contact (antiadhesive mechanisms) and to kill microbes on surfaces after contact (biocidal mechanisms). For better clarity of the text, we further discuss the above mechanisms in the following two subsections.

#### 2.3.1. Mechanisms Employed to Repel Microbes from Surfaces

Protein and microbial repellent/antifouling coatings prevent the attachment of proteins and microbes on surfaces but do not actively interact with or kill them. A typical repelling mechanism is corresponding to hydrophilic polymers that are coated or assembled on various surfaces, where they produce an aqueous interface, which repels microbes/foulants (when proteins or microorganisms are in proximity of the surface, water molecules are released from the interface, while polymer chains adopt more compressed conformations [37]). Therefore, the microbial adhesion strongly relies on the precise arrangements of the polymer chains into well-defined, often not easy to fabricate surface structures [76] that dictate the surface charge and chemistry, hydrophobicity, topography, roughness, stiffness (Figure 2 bottom) [77]. The repelling mechanism is based on various interactions of microorganisms with the surface they try to colonize. These interactions include the steric and the electrostatic repulsion (Figure 2 top).

Hydrophilic oligomers and polymers, such as oligoethylene glycol (OEG), polyethylene glycol (PEG), poly(poly(ethylene glycol) methacrylate) (PPEGMA) [78,79,80], take advantage of steric repulsion to repel microorganisms. For instance, PEG functionalized coatings form the hydration layer via hydrogen bonds and can prevent direct contact between microorganisms or proteins and surfaces with a certain efficiency. This repelling efficiency is low on surfaces of linear PEGs, in which molecules do not overlap, and higher on (star-shaped) PEG surfaces where molecules do interpenetrate and form a denser microstructure [81] (generally, a denser PEG microstructure can be obtained with shorter chains [82,83]). Moreover, the repelling efficiency is higher for microstructures made of PEG chains covalently bonded rather than less stable physioadsorbed chains [84].

The main disadvantage of the repelling mechanism of PEG-based polymers is its dependence on temperature [85] and time [76]. To overcome such drawbacks, zwitterionic polymers, i.e., neutral molecules that comprise both positive and negative charges, can be employed. The repelling mechanism based on zwitterionic polymers, such as poly(2-methacryloyloxyethyl phosphorylcholine) (PMPC) [86], poly(sulfobetaine methacrylate) (PSBMA) [87,88], poly(carboxybetaine methacrylate) (PCBMA) [87] or polybetaines [89] mainly operates by electrostatic repulsion, as multilayered surface structures are obtained via electrostatic interactions instead of hydrogen bonding. Owing to their great potential in withstanding protein adsorption [90] and to their specific molecular arrangements that allow a significant amount of water molecules to be bound on their surface, polymeric zwitterions are considered very attractive candidates for developing highly antiadhesive coatings [37,85]. Electrostatic repulsion can also be exploited to create antibacterial surfaces by employing anionic polymers. This is possible because bacterial cells are negatively charged (with only a few exceptions), and thus when approaching a negatively charged surface, electrostatic repulsion intervenes and prevents the bacterial adhesion [91,92].

At the end of this section, we briefly mention that polymers, such as polystyrene (PS), PC and polyethylene (PE), can also be used to fabricate extrinsic antimicrobial surfaces [93]. Such polymer chains can be directed to assemble into well-defined superhydrophobic micro-/nanostructures exhibiting low surface energy (Figure 2 top). It was demonstrated that only 2% of bacteria from a droplet adhered on such surfaces; less than 0.1% of bacteria remained attached on the surface after it was rinsed [93]. Bacterial cells can colonize a superhydrophobic structure only when the latter become fully wet and the air entrapped between the water droplets, and the structure is excluded [91].

#### 2.3.2. Mechanisms Employed to Kill Microbes on Surfaces

**Figure 3:**
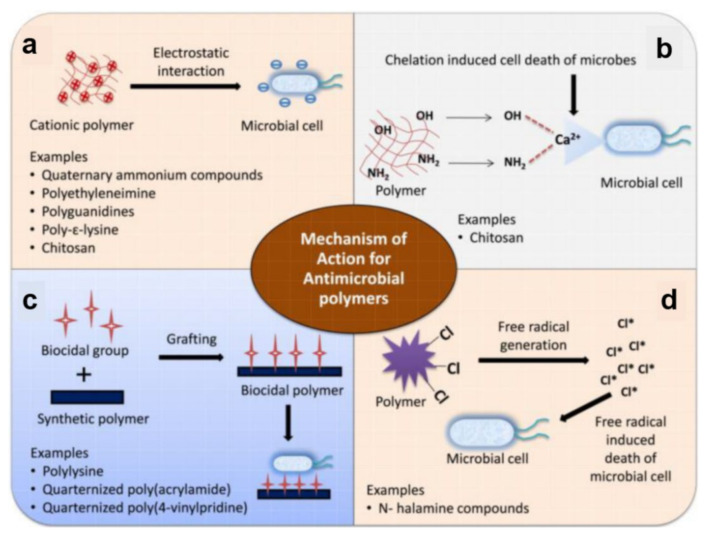
Schematic illustration of mechanisms of action of antimicrobial polymers based on electrostatic interactions (**a**), metal ion chelation (**b**), interactions with biocidal groups grafted on polymers (**c**), and release of halogen free radicals by N-halamine moieties (**d**). Adapted with permission from ref. [39].

Contact-killing surfaces express their biocidal properties when in contact with various microorganisms, including bacteria (Figure 3). Their major advantage is the possibility to hinder the rise of bacterial resistance, considering their non-specific mechanisms of action: physical damage of bacterial cells or ROS release [94]. For instance, cationic polymers, which contain both positive and hydrophobic functional groups [95], kill bacteria via electrostatic interactions between the cationic groups and negatively charged bacterial cells (Figure 3a). Because cationic polymers mimic the mechanism of action of antimicrobial peptides, the sequence through which they kill pathogens is the following: adsorption on the microbial cell surface, penetration into the cell wall, interaction with the cytoplasmic membrane, followed by irreversible damage of this structure, leakage of cytoplasmic components, such as electrolytes and nucleic acids, and, eventually, cell death. Unfortunately, antimicrobial surfaces that use cationic polymers are only suitable for short-term applications because their positive charges cause protein adhesion and accumulation of bacterial debris. They become inactive once covered by a (thick) layer of biomolecules and microbial fragments [96].

In comparison to cationic polymers, polymers based on N-halamines contain nitrogen atoms that covalently bind halogens, such as chlorine, bromine or iodine. These halogens can be slowly released into the environment and lead to generating ROS (Figure 3d) that can kill faster a broad spectrum of bacteria. In general, the biocidal activity of N-halamines can be restored using halogen-donor compounds (sodium hypochlorite/hypobromite) [97]. The mechanism of action of N-halamines has been described in two different ways: either the halogen is directly transferred to the microbial cell wall, followed by oxidation, or the dissociation into water is followed by diffusion over the bacteria. Afterward, the oxidative action of the halogen is directed to a biological receptor of bacterial cells (e.g., thiol or amino protein groups), causing metabolic inhibition or cell death. Therefore, in contrast to cationic polymers, the biocidal properties do not arise from the polymer itself but from the N-halamine functional group [39,98].

Other mechanisms used to kill microbes include chelation (Figure 3b) and interactions with biocidal groups grafted on polymer chains (Figure 3c). While the former mechanism is rather related to biopolymers, such as chitosan (not a topic of discussion in this work), the latter mechanism can be used to kill bacteria by grafting various biocidal groups on synthetic polymers, leading to biocidal polymers, such as quaternized poly(acrylamides) or quaternized poly(4-vinylpiridines). Of course, instead of grafting, various biocide moieties and disinfectants (metallic ions, quaternary ammonium compounds, antibiotics, antimicrobial peptides, or triclosan) can be loaded within inactive polymer matrixes to obtain biocide-releasing polymers. These can be further employed in the manufacture of leaching polymer surfaces [39] characterized by the mechanism of action depending on the released component [96]. Often, the antimicrobial agents can be released when certain physical, chemical, or biological stimuli are present. For example, temperature-responsive poly(N-isopropylacrylamide) surfaces can reduce bacterial adhesion or induce toxicity at particular temperatures. Instead, bio-responsive surfaces change their microstructure when exposed to enzymes or other constituents of the biological fluids. Similarly, pH-responsive surfaces are based on pH-dependent antimicrobial compounds, which are activated at low pH values and can be used for targeting acid-producing bacteria [40]. Due to the tremendous amount of data available in the literature on antimicrobial polymers, we further discuss within this review only the most recent antimicrobial polymeric structures assembled in/on coatings/surfaces.

## 3. Recent Antimicrobial Structures Assembled from Polymers on Surfaces

Several well-established polymer-based antimicrobial mechanisms are challenging researchers to employ synthetic polymers to generate efficient antimicrobial structures in various environments. A prominent category of such structures is represented by drug-loaded and gel-like structures [42,53,99,100,101,102]. Another equally important category of antimicrobial structures includes those obtained from polymeric systems assembled on specific surfaces [103,104,105,106,107,108,109,110,111,112,113,114,115,116]. In this work, we further review only the most recent antimicrobial structures falling within the last category.

We start by emphasizing that structures generating antimicrobial surfaces can be obtained through the assembly of both synthetic polymers and biopolymers, such as cellulose [117], chitin [118], chitosan [119], furcellaran [120], pectin [121], pullulan [122] or starch [123], etc. However, in this work, we present only polymer-based antimicrobial surface structures. Based on various antimicrobial mechanisms [75,76], these surface protecting structures rely on three categories of polymers. The first category comprises polymers employed to prevent the adhesion of microbes on surfaces rather than killing them by minimizing the protein adsorption (i.e., the biopassive antiadhesive class of polymers; see the antimicrobial mechanisms in Section 2.3.1). The second category is based on polymers, eventually covalently decorated with antimicrobial moieties and conjugates, that kill microbes when in contact (i.e., the bioactive antimicrobial class of polymers; see the antimicrobial mechanisms in Section 2.3.2). The third category is represented by antiadhesive polymers combined with various antimicrobial agents/polymers to exploit the synergistic effects against microbes and provide surface protecting structures with both microbe preventing and killing attributes.

### 3.1. Polymeric Structures Preventing the Adhesion of Microbes on Surfaces

Nowadays, it is of paramount importance to prevent various microorganisms from contacting, adhering and flourishing on specific surfaces in order, for example, to keep various medical devices/facilities uncontaminated [124,125,126,127], to generate efficient implants [105], or to avoid biocorrosion of marine-related applications [128]. Polymers can be employed to engineer films and coatings with tuned surface properties that can prevent the adhesion of microorganisms. One such polymer is PEG, a polyether that can be synthesized in various molecular weights and that can display a diversity of hierarchically ordered nanostructures at multiple-length scales, especially when incorporated in block copolymers [129,130]. This ability to adopt various highly ordered intrachain conformations endows PEG with protein repellent or attractive properties at low or high compressive loads, respectively [131]. It makes it a versatile material that can be used to efficiently fight microbes when coated on surfaces [103,104,105].

**Figure 4:**
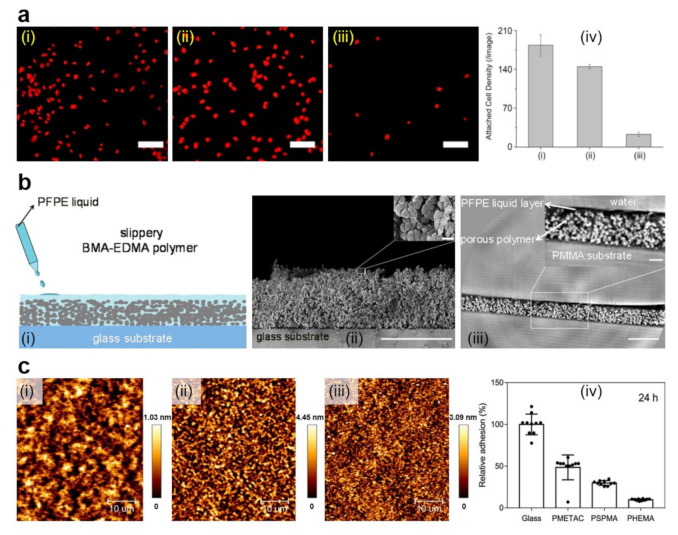
(**a**) Fluorescence images (i–iii) and quantification (iv) of *A. coffeaeformis* diatoms that succeeded to attach on the (i) untreated stainless steel, (ii) PDA-coated stainless steel, and (iii) PEG-based film. Each point is the mean of 60 counts on three replicate samples in (iv). Scale bars represent 50 μm. (**b**) Schematic representation of the fabrication of slippery P(BMA-*co*-EDMA) surface by infusion of the porous polymer with the PFPE fluid (i), scanning electron microscopy (SEM) images depict a cross-section and the porous morphology of the P(BMA-EDMA) surface (ii) and reconstructed X-ray propagation phase-contrast tomography image displaying the cross-section of the slippery P(BMA-EDMA) surface underwater (iii). Scale bars are 100 μm (ii,iii), 2 μm (ii) and 20 μm (iii) in the insets, respectively. (**c**) atomic force microscopy (AFM) height images depict PMETAC (i), PSPMA (ii), and PHEMA (iii) polymer brushes attached to the glass coverslips and their antiadhesive performance against *E. coli* compared to naked glass, after an incubation time of 24 h (iv). The relative adhesion of bacteria on the above substrates was normalized with respect to the adhesion on the glass substrate for 24 h. Adapted with permission from ref. [104] (**a**), ref. [107] (**b**) and ref. [139] (**c**).

Coating various surfaces with PEG can be mainly realized either by employing various direct deposition techniques [104,105] or through the grafting procedure, i.e., by covalently anchoring the polymer chains to surfaces as brushes [103]. The latter procedure has the advantage of facile tunning of the microbial adhesion through the control of polymer chain length and temperature at which experiments are performed [103] and can reduce the adhesion of microbes by several to tens of times (Figure 4a and Table 2) [104]. For example, PEG can be utilized to construct multilayered films on stainless steel, nylon, titanium oxide or silicon oxide substrates, previously covered with mussel-inspired polydopamine (PDA) [104]. Moreover, to target marine biofouling (i.e., problematic accumulation of diverse microorganisms, plants and/or algae on surfaces immersed in water that damages boats or various underwater constructions and devices) on the above-mentioned surfaces, PEG catechols, obtained by coupling the amine groups of 6-arm-PEG-amine and the carboxylic group of 3,4-dihydroxyhydrocinnamic acid [132], can be crosse-linked with PDA through catechol-catechol based interactions to become insoluble. The resulting multilayered films spin-cast from PEG catechol solution is not only stable under marine environments but also exhibits high resistance towards marine *A. coffeaeformis* (Figure 4a) [104]. Crosslinking process can further be employed to develop anticellular and bacterial repellent PEG-based coatings on bare and sandpapered titanium surfaces [105]. More exactly, nanofibers of PEG prepared via electrospinning can be coated on titanium surfaces and then rendered insoluble through using a photo-crosslinking agent. Results have shown that titanium surfaces covered with PEG nanofibers display enhanced antiadhesive properties against fibroblastic preosteoblasts and *S. epidermidis* compared to their counterpart blank surfaces [105].

Another class of polymers, known for their role in impeding the adhesion of microbes on surfaces, is represented by the zwitterionic polymers, such as polysulfobetaine (PSB) [133]. Moreover, methacrylate-based PSB (PSBMA) can be grafted on glass substrates by employing an atom transfer radical polymerization (ATRP) route and can lead to 10-15 nm thick brush-like structures of specific molecular conformations. These nontoxic structures can efficiently inhibit the adhesion of marine green algae, spores, sporelings and diatoms, such as *Navicula*, over various periods [106]. More recently, the microbe repellent properties of zwitterionic PSBMA were extended to *S. aureus* and *S. epidermidis* through the realization of thin brush-like films of PSBMA and PCBMA on polydimethylsiloxane (PDMS) surfaces [134]. PSBMA and PCBMA were covalently attached to PDMS substrates by employing a grafting method based on the exposure to UV light of the zwitterionic monomers in the presence of photoinitiators and crosslinking agents. Resulting PSBMA and PCBMA films decreased the adhesion of the above-mentioned bacteria under dry conditions by an order of magnitude [134]. A significant bacteria antiadhesive effect was also demonstrated for zwitterionic brushes grafted on brominated stainless steel and polymerized from 2-methacryloyloxyethyl phosphorylcholine and *N*-(3-sulfopropyl)-*N*-(methacryloxyethyl)-*N*,*N*-dimethylammonium betaine [135]. The thickness of these brushes was evaluated to be 53 and 132 nm, while the static water contact angle was measured to be only 14° and 11°, respectively.

The same ATRP reaction can be utilized along with photochemical grafting to grow protein-repelling zwitterionic brushes of poly [3-(methacryloylamino)propyl]dimethyl(3-sulfopropyl)ammonium hydroxide (PMPDSAH) onto indium thin oxide substrates [136]. Interestingly, the antifouling performance of PMPDSAH coated surfaces is outperforming that of the PEG surfaces [137]. Moreover, this polymer can be functionalized by a 1.9 nm thick metal–polyphenol coating to make it attractive to proteins and cells [138]. This is particularly important when targeting the spatio-selective functionalization of a specific surface. For instance, by patterning the PMPDSAH surface with a metal–polyphenol coating using a microcontact printing technique, surfaces displaying alternating hundred micrometers sized regions with fouling and antifouling properties can be fabricated [138].

Besides PEG and zwitterionic polymers, polyelectrolytes can also be used to create microbe repellent surfaces. Generally, the polyelectrolytes are structured within a multilayer configuration (also known as polyelectrolyte multilayer; PEM) through the layer-by-layer (LbL) deposition, assembly and/or functionalization procedures [140,141,142]. A relevant example of PEM structures was given by Schmolke and coworkers, who have combined poly(diallyldimethylammonium chloride) (PDADMAC) with poly(acrylic acid) (PAA) and also poly(allylamine hydrochloride) (PAH) with PAA. They have further adsorbed these systems on PDMS substrates by following an LbL assembling procedure. Resulting PDADMAC/PAA and PAH/PAA PEMs have demonstrated a decreased adhesion strength of *S. cerevisiae* on their surface [140]. Further adsorption of a pegylated PAA (PAA-g-PEG) on top of PAH/PAA or PDADMAC/PAA PEMs led to highly efficient coatings able to reduce the adhesion of microorganism cells up to two orders of magnitude.

Other microbe repellent polymers worth mentioning include poly(butyl methacrylate-*co*-ethylene dimethacrylate) (P(BMA-*co*-EDMA)) [107], poly(2-methyl-2-oxazoline) dimethylacrylate (PMOXDA) [108], negatively charged poly(3-sulfopropyl methacrylate potassium salt) (PSPMA) or neutral poly(2-hydroxyethyl methacrylate) (PHEMA) [139] (Table 2). While porous films of P(BMA-*co*-EDMA) can be infused with the liquid perfluoropolyether (PFPE) to fabricate slippery hydrophobic surfaces that efficiently prevent microbe adhesion and biofilm formation (Figure 4b) [107], PMOXDA can be copolymerized with different amounts of positively charged monomers of (2-(methacryloyloxy)ethyl)-trimethylammonium chloride (METAC) to lead to PMOXDA-*co*-PMETAC films displaying negative, neutral and positive surface zeta potential. Such films are then able to significantly reduce bacterial adhesion [108]. Moreover, PMETAC, PSPMA and PHEMA can be grafted onto glass surfaces by employing a surface-initiated ATRP reaction to develop polymer brushes adopting specific molecular conformations: surfaces are covered with a random few micrometer-sized PMETAC, micrometer-sized PSPMA and hundreds of nanometer-sized PHEMA features, respectively. Such brushes can control the surface-bacteria interactions and thus, can discourage the bacterial adhesion (Figure 4c) [139]. More details on polymers with antiadhesive properties and on strategies to prevent bacterial adhesion can be further consulted in the literature [37,38,78,79,80,86,87,88,89,141].

**Table 2:** Summary of polymeric systems and configurations that can be employed to prevent the adhesion of various microbes on surfaces. Following abbreviations were used: *Staphylococcus (S.)*, *Pseudomonas (P.)*, *Candida (C.)*, *Amphora (A.)*, *Saccharomyces (Sc.)* and *Escherichia (E.)*. Efficacy for biopassive systems refers to how many times fewer microbes were attached to the antiadhesive surfaces in a certain time as compared to their analogs.

Antimicrobial Polymer	Configuration/ Nanostructure	Dimension	Antimicrobial Mechanism	Efficacy	Microbe of Interest	Ref.
PEG	Nanofibers	167–184 nm diameter	Biopassive	~2–7 times	*S. epidermidis*	[105]
PEG	Brushes	2.8–23.7 nm length	Biopassive	~6 times ~6–8 times ~25 times ~4 times	*P. aeruginosa* *C. albicans* *S. epidermidis* *C. tropicalis*	[103]
PEG catechol	Multilayered films	5.2 nm thick	Biopassive	~8 times	*A. coffeaeformis*	[104]
PSBMA	Brushes	10–15 nm thick	Biopassive	~12 times ~7 times	Marine alga Ulva Diatom *Navicula*	[106]
PMPDSAH	Brushes	4.4 nm thick	Biopassive	-	Marine diatoms	[138]
PSBMA PCBMA	Thin-films	<25 µm thick	Biopassive	~10 times ~10 times	*S. aureus* *S. epidermidis*	[134]
PDADMAC/PAA, PAH/PAA	PEM structure	30–150 nm thick	Biopassive	~5 times	*Sc. cerevisiae*	[140]
PBMA-*co*-EDMA	Porous films	<100 µm thick	Biopassive	-	*P. aeruginosa*	[107]
PMOXDA-*co*- PMETAC	Thin-films	200 nm diameter, 230 nm thick	Biopassive	- -	*E. coli* *S. aureus*	[108]
PSPMA, PHEMA	Brushes	57 nm diameter, 43 nm thick	Biopassive	~9 times	*E. coli*	[139]

### 3.2. Polymeric Structures Employed to Kill Microbes on Surfaces

**Figure 5:**
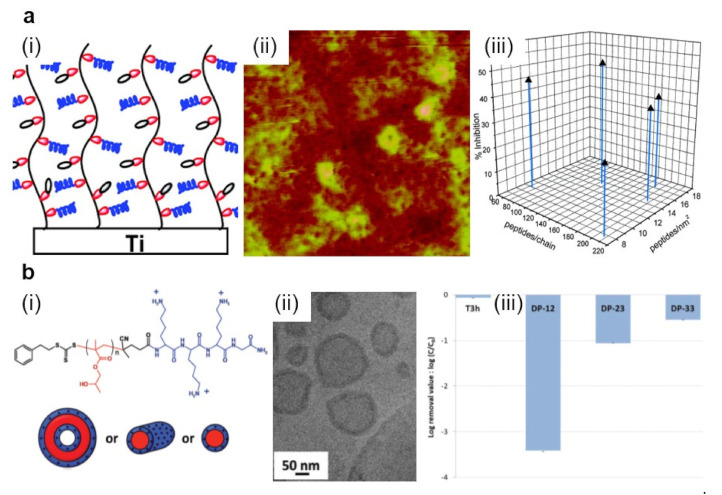
(**a**) Schematic representation (i) and AFM topography (ii) depict P(DMA-*co*-APMA) copolymer chains adopting brush-like conformations on titanium surfaces. Depending on the initial composition ratio between the DMA and APMA monomers and on the peptide density, P(DMA-*co*-APMA) copolymer exhibits more or less inhibitive effects against bacteria (iii). The size of the AFM image is 3 × 3 µm^2^. (**b**) Schematic representation (i) and a cryo-transmission electron microscopy (cryo-TEM) image (ii) depict spherical core–shell vesicles that could self-assemble from PLys-*b*-PHPMA block copolymer. The chemical structure of the copolymer is presented on top of (i). Experimentally observed structures are then able to act against *S. epidermidis* with high efficiency (iii). Log removal data were obtained after 3 h for the control reactor without any material (T3h) and the reactor with various PLys-*b*-PHPMA block copolymers. Adapted with permission from ref. [149] (**a**) and ref. [150] (**b**).

Bioactive polymers exhibit their antimicrobial attributes due to their intrinsic nature and/or their decorative biocidal moieties and conjugates incorporated into their backbone and/or side-chains [143,144,145]. An example of highly biocidal conjugates is given by the short antimicrobial peptides (AMPs), i.e., highly biocidal cationic units of gene-encoded peptide antibiotics possessing a low rate in driving antimicrobial resistance [146]. Generally, AMPs can be incorporated into polymeric device coatings and released to kill bacteria. Nonetheless, the time-limited antimicrobial effect is dictated by the elution of AMPs [147]. Instead, decorating polymers with AMPs might lead to augmented efficiency in targeting and killing the pathogens and can confer longer-term antibacterial properties while reducing the toxicity of the resulting systems against mammalian cells. In earlier studies, AMPs, such as Tet-213 (KRWWKWWRRC) [143] and other similar [144] were designed and tethered to poly-(*N,N*-dimethylacrylamide-*co*-*N*-(3-aminopropyl)-methacrylamide hydrochloride) P(DMA-*co*-APMA) copolymer chains that were grafted, in various molecular conformations and densities, from titanium surfaces (Figure 5a) [143,144]. The morphology of resulting surfaces consisted of different features exhibiting a roughness of about 6 nm. Moreover, higher copolymer brush densities rendered the resulting surfaces with more crowded AMPs and, thus, with better bactericidal properties [143].

More recently, Yu and coworkers have modulated the functionality of AMPs along with that of polymer brushes to generate high antimicrobial peptide potency that could be used in developing infection-resistant implant surfaces [148]. Their results revealed that the antimicrobial activity of brush coatings tethered with AMPs depended on the polymer brush chemistry (which has an impact on changes in the secondary structure of the AMPs) as well as on the AMP molecular conformations (which determine the density of AMPs on the polymer brushes and their microstructure) [148]. The microstructure of AMPs is further important because it regulates the rather unwanted interactions between peptides and biomolecules, such as blood proteins. An approach to eliminate such interactions, and thus, to allow peptides to attach effectively to the negatively charged bacterial surface, is to actually covalently connect few units of helical antimicrobial peptides with radial amphiphilicity into short polypeptides with a hydrophobic helical core and a charged shell [149]. Consequently, the resulting polypeptides can adopt peculiar conformations and form nanostructures exhibiting one to two orders of magnitude enhancement in their antimicrobial activity against both Gram-positive and Gram-negative bacteria [149].

Enhanced antimicrobial activity was further noticed for similar spherical nanostructures made of polylysine-*b*-poly(2-hydroxypropyl methacrylate) (PLys-*b*-PHPMA) block copolymer [150]. These nanostructures were created via polymerization-induced self-assembly of HPMA by employing PLys as the macrochain transfer agent and are comprised of a PHPMA core and a PLys shell. For instance, simple vesicles or vesicles with rather few and short branched worms formed when long or shorter PHPMA polymer chains were employed, respectively (Figure 5b). The obtained spherical nanostructures exhibited significant antimicrobial properties in thin-film membranes. This was possible due to the positively charged nature of PLys chains forming the shell of the spherical particles [150].

Another widely-used biocidal moiety that can be tethered to various polymer chains to render them antimicrobial is the quaternary ammonium (QA) salt, a disinfectant used already for many years to kill microbes, such as bacteria, yeasts or molds [151]. QA moiety can be attached to various copolymers made of polymethylhydrosiloxane (PMHS) and PDMS via a quaternization procedure based on 1-iodooctane. This procedure leads to crosslinked QA-functional polymers containing different concentrations of QA moieties that can generate highly homogeneous films. The latter can be then optimized with respect to the moisture curability and used to correlate the QA concentration with the biocidal activity toward the marine bacterium *Cellulophaga lytica* and algae *Navicula incerta* [151]. Optimized samples exhibit about 80% in biofilm retention and 90% reduction in biofilm growth for the two microbes, respectively. Similarly, QA-based antimicrobial coatings can be prepared by dip-coating the surface of interest directly into a solution made of amphiphilic poly((dopamine methacrylamide)-(methoxyethyl acrylate)-dodecyl QA) (P(DMA-MEA-DQA)) containing various amounts of antimicrobial dodecyl QA, hydrophobicity tuning methoxyethyl and immobilizing catechol groups [109]. The resulting antimicrobial polymer films display a smooth morphology comprised of dodecyl chains localized rather at the air-surface interface and with the phenyl groups of the catechols oriented with respect to the substrate surface. Instead, the most hydrophobic films made of polymers containing no methoxyethyl side-chains were comprised of polymeric domains of an average size of hundreds of nanometers that exhibited high surface roughness. All films proved themselves highly biocidal against various microbes, inclusively due to their adhesive functionality of catechol groups that have prevented the leaching of polymers [109].

QA moiety can be further attached to polymers, such as polyurethane (PU) [145] and poly(2-(dimethylamino)-ethyl methacrylate-*co*-methyl methacrylate) (P(DMEMA-*co*-MMA)) [152]. In the first case, QA salt moieties and hydroxyl groups are introduced to the backbone of the soybean oil-based polyols and then are reacted with diisocyanate monomers to obtain PUs. PU coatings containing more QA salt moieties exhibit the best antibacterial activity by killing about 95% of bacteria [145]. In the second case, a solution of partially quaternized P(DMEMA-*co*-MMA) mixed with ethylene glycol dimethacrylate (EGDMA) and photoinitiator 2-hydroxy-4-(2-hydroxyethoxy)-2-methylpropiophenone (HHMP) is spin-cast on glass slides and cured under UV light to generate a semi-interpenetrating network (SIPN) of P(DMEMA-*co*-MMA) and polymerized EGDMA [152]. This QA-garnished polymer-based coating not only displays strong bacteria-killing properties but can prevent the biocidal QA moieties from leaching.

**Figure 6:**
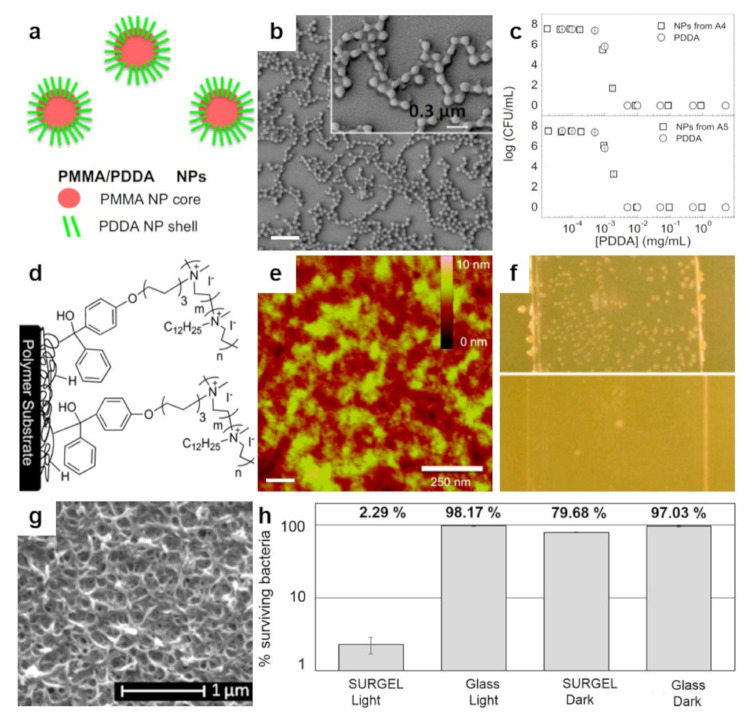
(**a**,**b**) Schematic representation (**a**) and SEM morphology (**b**) of the core–shell PMMA/PDDA NPs assembled at low ionic strength. The scale bar represents 1 µm. (**c**) Antimicrobial activity of NPs against *E. coli*. Representation of cell viability (log10 of colony-forming units CFU/mL) for *E. coli* with respect to the concentration of free PDDA or PDDA in the PMMA/PDDA NPs exhibiting sizes of 112 nm and 164 nm in diameter, respectively. (**d**) Schematics depict the covalent attachment of the benzophenone-based PEI copolymer to various surfaces and plastics. (**e**) Tapping-mode AFM height image depicts the surface of a thin-film of benzophenone-based PEI copolymer after sonication. (**f**) Digital images of a control glass substrate (top) and of a glass substrate modified with benzophenone-based PEI copolymer (bottom) sprayed with *S. aureus* and incubated for 24 h. (**g**) SEM micrograph depicts the surface of a porphyrin-based SURMOF after crosslinking and subsequent treatment with EDTA solution. (**h**) Antibacterial activity of the porphyrin SURGEL thin-films against *E. coli* using the LIVE/DEAD BacLight bacterial viability kit. Adapted with permission from ref. [153] (**a**–**c**), ref. [110] (**d**,**e**) and ref. [159] (**g**,**h**).

The fact that ammonium-based polymers are capable to efficiently kill bacteria was recently further demonstrated by Sanches and coworkers, who generated core–shell NPs by decorating the poly(methyl methacrylate) (PMMA) NPs with antimicrobial poly(diallyldimethylammonium chloride) (PDDA) via emulsion polymerization reaction (Figure 6a) [153]. Resulting cationic NPs, self-assembled under low ionic strength conditions (Figure 6b), can access the inner layers of the cell and its membrane through the antimicrobial action of the PDDA shell and, thus, can kill microbes very efficiently (Figure 6c). The killing efficiency is nonetheless depending on the hydrophobic-hydrophilic balance of PDDA, as well as on the type of microbe, possibly due to the microstructural differences of the microbial cell walls [153]. More recently, PDDA/PMMA core–shell NPs were further optimized and utilized to fabricate antimicrobial coatings on substrates of interest by drop or spin casting [154]. Deposited hydrophilic coatings exhibited contact angles depending proportionally on the amount of PDDA in the NPs and reduced bacteria by about 7 logs.

A distinguished class of polymers that can be successfully used to generate antimicrobial structures on surfaces is represented by the cationic polymers. The main representants of this class of polymers are polyethylenimines (PEIs). The antimicrobial efficiency of PEIs, deriving from their cationic character, depends on optimizing their chemical structure [155]. For example, to enlarge their capability to perforate the hydrophobic membrane of bacteria, linear or branched PEIs with different molecular weights need to be synthesized [156]. While both types of PEIs possess enhanced antibacterial activity against *S. aureus*, only linear PEIs can induce depolarization of the bacterial membrane. Furthermore, PEIs can be N-alkylated with quaternary amino functional groups, such as hexyl, octadecyl or dodecyl, just to name a few, and rendered antimicrobial when deposited on surfaces or incorporated into NPs [155]. Besides N-alkyl, benzophenone can also be used to decorate PEIs. N-alkylated and benzophenone-based PEIs synthesized from poly(2-ethyl-2-oxazoline) (PEOX) can be then attached by photo-crosslinking to various surfaces, such as cotton, silicon oxide, or various other polymers, to fabricate leaching-free antimicrobial coatings for textile and plastic materials (Figure 6d) [110]. Exhibiting a morphology comprised of random, tens of nanometers sized features of the roughness of less than one nanometer (Figure 6e), resulting PEI-based coatings are capable to kill more than 98% of *S. aureus* or *E. coli*. (Figure 6f).

Besides PEIs, various other cationic polymers exhibiting important antimicrobial properties were reported. They were recently thoroughly reviewed by Alfei and Schito, and therefore, additional information on this topic can be found elsewhere [157]. We would only like to additionally emphasize the existence of a new water-soluble cationic copolymer synthesized from 4-ammoniumbuthylstyrene hydrochloride and reported only very recently [158]. This copolymer was shown to be able to display a rapid non-lytic bactericidal activity and thus, to be capable of acting against several bacteria, including *E. coli*, *Acinetobacter baumannii* and *Stenotrophomonas maltophilia*.

Antimicrobial polymers can be further synthesized by incorporating in their chemical structure the N-halamine, a biocidal moiety capable of almost instant and total sterilization over a broad spectrum of microorganisms [160,161]. Importantly, N-halamine polymers do not form toxic products and do not release halogen unless they are in contact with bacteria [162]. The synthesis of N-halamine-based polymers and grafted copolymers are cheap and rely on employing 4-(alkyl acryloxymethyl)-4-ethyl-2-oxazolidinones and commercial monomers or polymers (the latter are well-known for their efficacy to kill bacteria in both granular forms and as surface, coatings covering glass or plastics [163]). Alternatively, any inert polymer, including bilayers of PS functionalized with a top surface layer of poly(styrene-*b*-tert-butyl acrylate) (PS-*b*-PtBA), can be employed to generate the desired density of chemical groups containing amine bonds grafted from PS surfaces (i.e., PS/PS-PAA) and can be further chlorinated to N-halamine [164]. Resulting N-halamine polymeric surfaces are highly biocidal against *S. aureus* and *E. coli*. Unfortunately, such antimicrobial systems are not very stable and need re-chlorination, as a significant part of the chlorine is lost upon UV irradiation [164].

Other N-halamine polymer precursors of cationic homopolymer poly((3-acrylamidopropyl) trimethylammonium chloride) (PCHP) and of anionic homopolymer poly(2-acrylamido-2-methylpropane sulfonic acid sodium salt) (PAHP) [111] or of poly[5,5-dimethyl-3-(3′-triethoxysilylpropyl)-hydantoin] (PSPH) [165] can be synthesized and coated onto cotton fabrics or mesoporous molecular sieves via LbL deposition or grafting techniques. The resulting N-halamine coatings can be further rendered biocidal upon their exposure to household bleach. Both concepts lead to chlorinated coatings of well-defined roughness that can inactivate 100% of *S. aureus* and *E. coli* [111,165], with significant log reductions within the first minute of contact [165]. Again, washing of the fabrics coated with N-halamine polymers is accompanied by a reduction of chlorine. This drawback is compensated by the fact that coated fabrics produce no irritations to rabbit skin, displaying thus important potential towards future biomedical applications [111]. More recently, the process of chlorine bleaching at different bleach concentrations on tape was further employed to transform polypyrrole (PPy) into N-halamines and to develop highly efficient antimicrobial coatings on stainless steel by taking advantage of the electrochemical deposition process [166].

At the end of this section, we note other peculiar polymers, such as porphyrins, which can be integrated as constituents into a surface anchored metal–organic framework (MOF). The resulting composition can be deposited on a substrate and crosslinked, leading to SURMOF (Figure 6g). The latter can be transformed by a treatment with ethylenediaminetetraacetic acid (EDTA) solution into a metal-free antimicrobial polymer-based coating abbreviated SURGEL that demonstrates significant antimicrobial activity by killing more than 97% of some bacteria (Figure 6h). This is possible due to ROS generation when thin polymer films based on porphyrin are exposed to visible light [159]. While a summary of results obtained on bioactive polymers exhibiting antimicrobial properties is presented in Table 3, additional details can be found in comprehensive scientific papers available in the literature [146,167,168].

### 3.3. Polymeric Surface Structures Exhibiting Microbe Antiadhesive and Killing Properties

An interesting strategy against the accumulation of microbes on surfaces relies on combining the antiadhesive and antimicrobial properties of polymers to develop more complex bifunctional surface systems capable of both repelling and killing microbes. This strategy can be implemented by conjugating antiadhesive polymers with various antimicrobial (biocidal) moieties or even polymers. A relevant example was given a decade ago by Muszanska and coworkers, who have synthesized a triblock copolymer with a central polypropylene oxide (PPO) block and two terminal antiadhesive PEG segments under the name of Pluronic F-127 (PF-127) [169]. At the telechelic groups of the PEG chains, they have further covalently attached the antimicrobial enzyme lysozyme conjugate. This triblock copolymer led to structures comprised of one or two lysozyme molecules per each PF-127 polymer chain that could adsorb on a hydrophobic surface by adopting a brush-like molecular conformation. Surfaces coated with such brushes showed both antiadhesive and antimicrobial properties. Intriguingly, the structures with less lysozyme coverage obtained from a mixture of unconjugated PF-127 and PF-127-lysozyme conjugates were more bactericidal than brushes realized only from PF-127-lysozyme conjugates [169].

**Figure 7:**
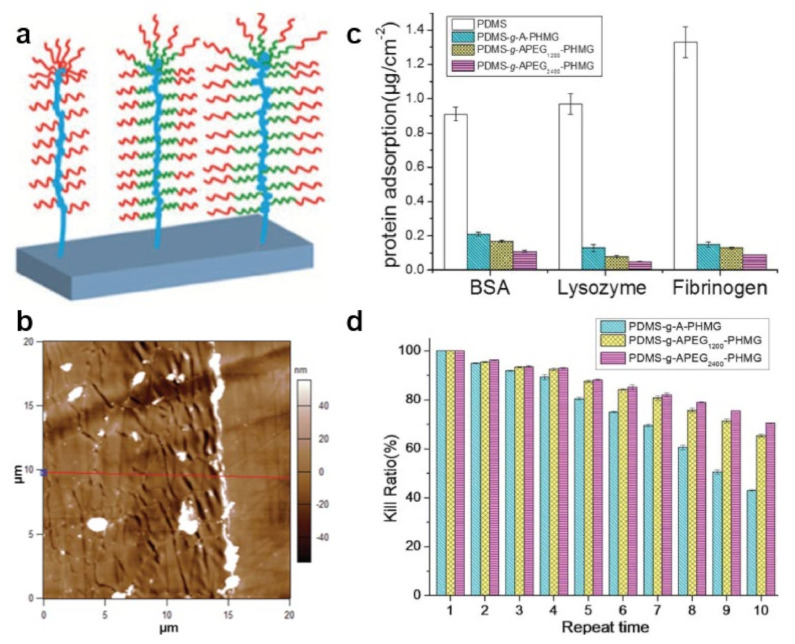
(**a**) Schematics of the APEG_2400_–PHMG bottlebrushes grafted from a polymer-covered substrate. (**b**) AFM topography image depicts the surface morphology of the APEG_2400_–PHMG coating (on the left side) and of pristine PDMS (on the right side). The red line corresponds to a height cross-section used to evaluate surface roughness. (**c**,**d**) Protein adsorption (**c**) and long-term reusable antibacterial properties (**d**) of pristine PDMS and of PDMS surfaces coated with allyl terminated PHMG or with APEG–PHMG. The antibacterial properties were tested against *P. aeruginosa*. Adapted with permission from ref. [171].

Bifunctional brushes adopting a bottle-like conformation were further designed and developed from a block copolymer obtained through the conjugation of antimicrobial polyhexanide (PHMB) with allyloxy PEG of both low and higher molecular weight. APEG_1200_-PHMB and APEG_2400_–PHMB copolymers assembled into 25 nm-thick bottlebrush nanostructures when grafted, via surface-initiated polymerization, from silicone rubber surfaces [170]. Nanostructures assembled from both copolymers showed excellent antimicrobial properties against Gram-negative and Gram-positive bacteria, with the emphasis that the APEG_2400_–PHMB coating exhibited improved antiadhesive properties, most probably due to more abundant PEG units incorporated in its chemical structure [170]. A similar strategy was used to synthesize block copolymers with the same amount of PEG units but attached to an antimicrobial polyhexamethylene guanidine (PHMG) block [171]. As expected, the nanostructures obtained by grafting these block copolymers from silicone surfaces are bifunctional 20 nm thick bottlebrushes displaying a “crinkled” morphology. In this case, too, the APEG_2400_–PHMG coating could inhibit the adsorption of proteins and kill bacteria more efficiently than its counterpart (Figure 7) [171].

Antiadhesive PEG can further be used, along with a cationic PC combined either with tethering or with an adhesive functional block, to synthesize V- and S-shaped triblock copolymers by placing the tethering block centrally or at the end, respectively [172]. While the surfaces coated with V-shaped polymer exhibited antibacterial properties but without being able to prevent microbial adhesion, the surfaces coated with S-shaped polymer exhibited strong antibacterial and antiadhesive attributes [172]. In comparison, linear PEG-*b*-PC diblock copolymers were also reported to exhibit both antiadhesive and antimicrobial properties when grafted onto PDA-covered silicone rubber surfaces [173]. Furthermore, if PC is replaced with cationic antimicrobial polypeptides, PEG-*b*-polypeptide diblock amphiphilic polymer chains with both antimicrobial and antiadhesive segments can be obtained via ring-opening polymerization (ROP) of N-carboxyanhydrides [174]. These polymers can be then grafted onto the PDMS surface via surface-induced polymerization to form bottlebrush nanostructures able to repel and kill microbes, such as *E. coli*, *P. aeruginosa* or *S. aureus*.

An efficient approach to combine antiadhesive and antimicrobial (polymeric) entities is based on the LbL deposition technique. For example, as synthesized hydantoinyl acrylamide-*co*-trimethyl-2-methacryloxyethylammonium chloride and hydantoinyl acrylamide-*co*-2-acrylamido-2-methyl-1-propanesulfonic acid polyelectrolytes can be deposited one layer at a time onto polypropylene (PP) fabrics either as single or as multilayers [175]. Resulting copolymer-based layered structures, when embedded into dilute sodium hypochlorite solution, can reduce microbes by about 6 logs within the first two minutes of contact [175]. To increase the stability of multilayered polyelectrolytes, it looks more appealing to “click” the antiadhesive polymer with another antimicrobial polymer. For instance, Yang and coworkers combined antiadhesive azido-functionalized polyethylene glycol methyl ether methacrylate-based (PEGMA) polymer chains with antimicrobial alkynyl-functionalized 2-(methacryloyloxy)ethyl trimethyl ammonium chloride-based (PMETA) polymer system via a click-based LbL technique [112]. Practically, by repetitive deposition of a layer of the antiadhesive polymer on top of a layer of the antimicrobial polymer, multilayered polymeric coatings were obtained. These coatings were demonstrated to be not only resistant to bacterial adhesion but also bactericidal to marine microorganisms. One advantage of such multilayered structures is given by their tunable antimicrobial efficiency that depends on the number of polymer layers [112]. Interestingly, AFM studies conducted on the topography of such polymeric films revealed that the surface roughness decreased by almost 100% when increasing the number of polymer bilayers from 1 to 11, indicating a more compact coating structure for the thicker films [112].

**Figure 8:**
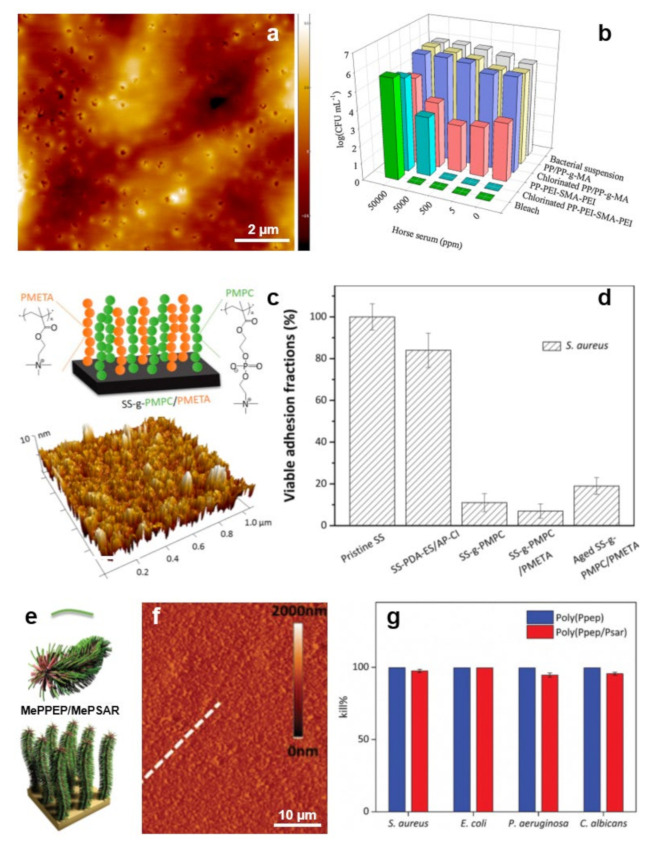
(**a**) AFM topography image depicts the morphology of the as-prepared PEI/SMA/PEI surface. (**b**) Antimicrobial properties of non-chlorinated and chlorinated PEI/SMA/PEI systems with respect to their analogs. (**c**) Schematic representation (top) and surface morphology, as revealed by AFM (bottom), of the PMPC/a-PMETA binary polymer brushes. (**d**) Percentage of the adhered *S. aureus* cells on the pristine and PMPC/a-PMETA surfaces compared to their analogs before and after aging, after exposure to the bacterial suspension in artificial seawater for 4 h. (**e**,**f**) Schematics (**e**) and AFM topography image (**f**) depict MePPEP/MePSAR binary brushes on a solid surface. White broken line in (**f**) was used to estimate the film roughness. (**g**) Contact bactericidal activity of the MePPEP and MePPEP/MePSAR coatings against various microbes. Adapted with permission from ref. [176] (**a**,**b**), ref. [177] (**c**,**d**) and ref. [178] (**e**–**g**).

Coatings with compact structures simultaneously exhibiting antiadhesive and antimicrobial properties can also be obtained when spin casting, on top of PP/PP-graft-maleic anhydride hot-pressed coupons, branched PEI and styrene-maleic anhydride (SMA) copolymer in a PEI/SMA/PEI configuration [176]. The resulting structure exhibiting pores of about 100 nm in diameters (Figure 8a) was formed from hydrophobic styrene subunits, cationic primary amine groups with intrinsic antimicrobial properties and chlorinated N-halamine-based groups exhibiting enhanced antimicrobial attributes (Figure 8b). Experiments have revealed no evidence of *E. coli* adhesion on the PEI/SMA/PEI-coated surface [176].

Often, the bilayer/multilayer film configurations are unable to allow full percolation on the top surface of both the antiadhesive and antimicrobial polymer components. This inconvenience can be avoided by using peculiar grafting techniques. Here, polymer systems with antiadhesive properties can be grafted from a brush-like substrate displaying antimicrobial properties [179], or both antiadhesive and antimicrobial polymer components can be grafted from the same substrate, leading to surfaces comprised of the mixed brush “forests” [177]. The first approach can deliver bifunctional materials comprised of antiadhesive poly(oxonorbornene)-based zwitterions grafted onto the brush-like polymer network of antimicrobial cationic poly(oxonorbornene). The resulting structures are 30–40 nm thick while displaying a roughness of 3–4 nm. Their morphology consists of 5 nm deep pores randomly and homogeneously distributed over the whole surface [179] (more details on how polyzwitterions can be grafted onto a carpet of polycationic antimicrobial polymers can be found elsewhere [180]). The second approach, based on specific surface modifications, favors the assembly of zwitterionic poly(2-methacryloyloxyethyl phosphorylcholine) (PMPC) and alkynyl-modified cationic poly(2-(methacryloyloxy) ethyl trimethylammonium chloride) (a-PMETA) binary polymer brushes onto PDA-anchored stainless steel surfaces through thiol-ene and azide-alkyne graft polymerizations, respectively (Figure 8c). Resulting PMPC/a-PMETA binary polymer brushes not only display a smooth surface with a roughness of ~1.2 nm but are also highly stable in seawater. More important, they endow the stainless steel surfaces with antiadhesive and antimicrobial attributes, especially against Gram-positive *S. aureus* (Figure 8d) and Gram-negative *Pseudomonas* sp. bacteria [177].

Antimicrobial a-PMETA can also be coupled with antiadhesive alkyne-functionalized poly(N-hydroxyethyl acrylamide) (a-PHEAA) and then further grafted from stainless steel to obtain bifunctional brushes able to fight against Gram-negative *E. coli* and Gram-positive *S. epidermidis* [181]. Similarly, antiadhesive methacrylate-ended polysarcosine (MePSAR) and antimicrobial cationic methacrylate-ended polypeptides (MePPEP), synthesized via ROP of N-carboxyanhydrides, can be assembled on PDA coated substrates via grafting initiated under UV irradiation. The resulting MePPEP/MePSAR binary brushes can display, on flat surfaces, a roughness as high as 44 nm and can exhibit highly antiadhesive and antimicrobial properties against several microbes (Figure 8e–g) [178]. Relevant information of this subsection is summarized in Table 4.

## 4. Antimicrobial Surface Structures Developed from Polymer Blends

**Figure 9:**
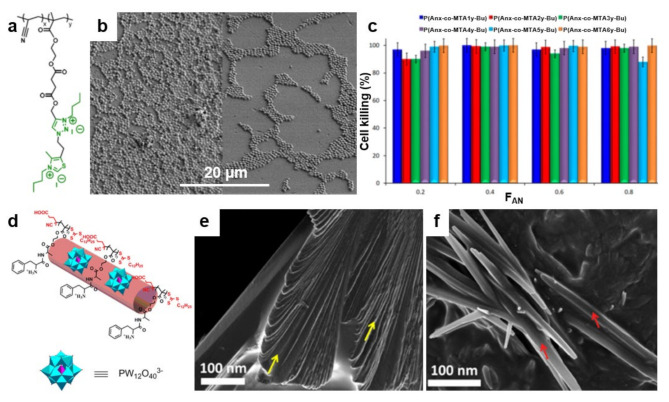
(**a**) Chemical structure of quaternized P(AN-*co*-MTA) acrylonitrile-based copolymers. (**b**) Field emission SEM micrographs depict *S. aureus* on PAN films in the absence (left) and in the presence (right) of a P(AN-*co*-MTA) antimicrobial copolymer after 2 h of contact. (**c**) The percentage of cell-killing for *C. parapsilosis* microorganism when in contact with antimicrobial films. (**d**) Schematic representation of the cationic/anionic P(Boc-FA-HEMA)/POM amphiphilic supra-assemblies. (**e**,**f**) Field emission SEM micrographs depict the morphology of P(Boc-FA-HEMA) before (**e**) and at 90 min after the addition of POM (**f**). The morphology of P(Boc-FA-HEMA) is comprised of individual sheets indicated by yellow arrows in (**e**). These sheets dissolve after the addition of POM and lead to forming nanorods, indicated by red arrows in (**f**). Adapted with permission from ref. [113] (**a**–**c**) and ref. [187] (**d**–**f**).

Recently, an alternative concept to develop antimicrobial structures on surfaces has emerged. Researchers have observed that blending polymers possessing antiadhesive or antimicrobial properties leads to peculiar nanostructures and chain conformations that enhance the above attributes when fighting microbes (Table 5). This concept can be implemented in both thin film [113,182] and brush configurations [183] by blending, for instance, an “inert” polymer with a copolymer deriving from it but decorated with antimicrobial moieties. Such a procedure facilitates an efficient control of the resulting film microstructure and thus, favors the optimization of the final antimicrobial properties. In this sense, it is possible to blend thermally stable polyacrylonitrile (PAN) with antimicrobial methacrylic copolymers bearing cationic moieties with 1,3-thiazolium and 1,2,3-thiazolium side-chain groups P(AN-*co*-MTA) (Figure 9a). While the morphology of the PAN/P(AN-*co*-MTA) films is smooth and homogeneous with rather few irregular pores, the surface wettability depends on the chemical compositions of the copolymers, i.e., on their flexibility and polarity, rendering PAN/P(AN-*co*-MTA) films with good antimicrobial properties (Figure 9b) [113]. Moreover, by increasing the density of the positive charges in such blend systems, the biocidal capacity can be increased to almost 100% cell-killing efficiency of bacteria and yeasts (Figure 9c).

Biocidal surfaces with high positive charge density were further fabricated from polymer blends containing PS and block copolymers comprised of PS and an antimicrobial block poly(4-(1-(2-(4-methylthiazol-5-yl)ethyl)-1H-1,2,3-triazol-4-yl)butyl methacrylate) decorated with either methyl or butyl groups (PS-*b*-PTTBM) [182]. By employing the breath figures approach (see details on surface relief patterns elsewhere [184]), porous yet ordered PS/PS-*b*-PTTBM films with narrow pore size distribution and low content of antimicrobial moieties were obtained. These structures showed increased antibacterial efficiencies against microbes, such as bacteria and fungi [182].

In analogy to biocidal polymers, polymers with protein repellent properties (e.g., PEG) can also be blended with other “inert” polymers (e.g., PAA) to control the adhesive properties of the former. This can be done by developing brush-like films of controlled PEG/PAA ratio and by utilizing PEGs of optimized molar mass [183]. Simply, by dip-coating a gold surface into a solution containing both polymers, mixed PEG/PAA polymer brushes are obtained. When PEG content is low, brushes exhibit higher affinity towards proteins, and the latter can irreversibly adsorb on the surface. Instead, when the PEG content is increased to over 25 PEG units per squared nanometer, the adsorption process becomes completely reversible, i.e., the adsorbed proteins can also be removed [183]. Moreover, PEG can be blended with antimicrobial biopolymers, such as chitosan, to obtain electrospun bifunctional nanofibers able not only to kill bacteria but also to promote osteoconductive activity [185].

Furthermore, one can blend microbe repellent polymers with microbe-killing polymers to obtain polymeric structures exhibiting both biopassive and bioactive attributes. This was demonstrated recently by Muszanska and coworkers, who have fabricated bifunctional brush-like coatings by dip-coating silicone rubber surfaces into solutions containing a mixture of antiadhesive PF-127, PF-127 conjugated with AMPs and PF-127 decorated with arginine-glycine-aspartate (RGDs) peptides [186]. The resulting brushes possess not only antiadhesive and antimicrobial attributes but are also capable of supporting mammalian cell growth. Because bacteria are significantly smaller than the mammalian cells and a limited number of RGDs may be enough to favor cell adhesion without notably affecting the adhesion of bacteria, a balanced optimization of the ratio of the three polymeric components can endow these brushes with antimicrobial attributes of either a more repelling or a predominant killing nature [186].

Finally, it is possible to blend one antimicrobial polymer with another antimicrobial polymer to create more complex structures with strengthened biocidal attributes able to kill microbes aggressively. This was recently demonstrated by Datta and coworkers, who blended a peptide-based antimicrobial poly(Boc-Phe-Ala-oxyethyl methacrylate) P(Boc-FA-HEMA) with antimicrobial polyoxometalate (POM) and obtained cationic/anionic P(Boc-FA-HEMA)/POM amphiphilic supra-assemblies (Figure 9d) [187]. These supra-assemblies are a result of the electrostatic attraction between the P(Boc-FA-HEMA) and POM, which transforms the cationic β-sheets (Figure 9e) to cationic multivalent nanorods (Figure 9f). Interestingly, while the peptide-based polymers kill microbes by disrupting their membrane, multivalent nanorods seem to also induce free radical-mediated cell damage, thus amplifying the antimicrobial efficacy [187].

**Table 5:** Summary of the polymeric blends that can be used to fight microbes on surfaces.

Blend/Composite	Configuration/ Nanostructure	Dimension	Antimicrobial Mechanism	Efficacy	Microbe of Interest	Ref.
PS/PS-*b*-PTTBM	Porous films (breath figures)	5–11 µm diameter	Bioactive	*99.99%* *90%*	*S. aureus* *C. parapsilosis*	[182]
PAN/P(AN-*co*-MTA)	Thin films	-	Bioactive	*~100%* *~100%* *~100%*	*S. aureus* *P. aeruginosa* *C. parapsilosis*	[113]
PEG/chitosan	Nanofibers	294 nm diameter	Bioactive	*-*	*S. epidermidis*	[185]
PF-127/PF-127-AMPs/PF-127-RGDs	Blend brushes	-	Biopassive + bioactive	*-* *-*	*S. epidermidis* *P. aeruginosa*	[186]
P(Boc-FA-HEMA)/POM	Multivalent nanorods	10–20 nm wide, a few µm long	Double bioactive	*100%* *-*	*E. coli* *B. subtilis*	[187]

## 5. Antimicrobial Surfaces Generated from Polymer-Based Nanocomposites

Antimicrobial properties of polymers can be significantly reinforced by exploiting various synergistic effects appearing when mixing these polymers with other materials displaying biocidal properties. For instance, antimicrobial polymers, such as PEG [114], PEI [188], zwitterionic PCBMA [189] or cationic poly(2-(tert-butylaminoethyl) methacrylate) (PTBAM) [190] can be utilized along with biocidal metallic NPs (Ag [74], copper/Cu [191], etc.) or carbon-based nanostructures [97] to develop highly biocidal nanocomposites (Table 6). Metallic NPs are increasingly used as an alternative to antibiotics for a myriad of purposes, including antibacterial coatings for implantable devices, to prevent infections [41]. Their main advantage consists in the possibility to overcome the ability of bacteria to develop resistance due to the fact that most of the antibacterial mechanisms (see more details in Section 2.2) are simultaneous and do not allow a bacterium to develop mutations on multiple genes [41]. Furthermore, to expand the biocompatibility and bioavailability of inorganic NPs, their further coating with various functional polymers can be performed (coating of NPs increases the oxidation stability of the core and diminishes particle aggregation in solution [192]). In earlier studies, the biologically inert silica-based core was coated with a layer of bactericidal polymers, but more recently, the attention was focused on the synergistic action of bactericidal metal-based NPs along with polymers with inherent antimicrobial activity [42] (the latter can easily integrate metal NPs of different shapes and sizes owing to their adjustable surface, morphology and porosity).

**Figure 10:**
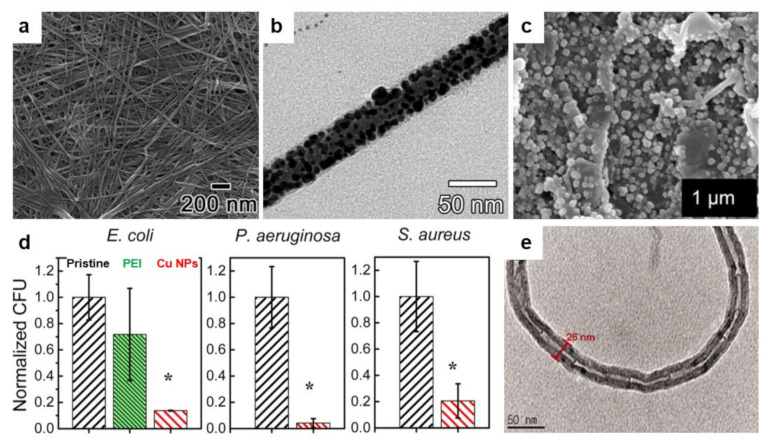
(**a**,**b**) Field emission SEM (**a**) and TEM (**b**) micrographs depict biocidal Ag NPs embedded in antimicrobial PTBAM nanofibers. (**c**) SEM micrograph depicts a membrane with bound Cu NPs after its sonication for 5 min in deionized water. (**d**) Number of attached live bacteria on pristine, PEI alone, and Cu NPs-based membrane for Gram-negative *E. coli* and *P. aeruginosa* and Gram-positive *S. aureus* bacteria. Asterisks (*) are emphasizing statistically significant differences observed between the functionalized and pristine membranes. (**e**) TEM micrograph depicts the morphology of PDMEMA-MWNT nanocomposite containing almost 25 wt % of PDMEMA. Adapted with permission from ref. [190] (**a**,**b**), ref. [188] (**c**,**d**) and ref. [195] (**e**).

Ag NPs, typically displaying a diameter in the range of 1 to 100 nm, were demonstrated to be highly efficient antibacterial agents [197]. Thus, their further combination with antimicrobial polymers is expected to increase the bacteria-killing efficiency. Because Ag NPs may possess various shapes and surface properties, the corresponding bacteria-killing mechanism is rather complex and may occur following multiple pathways (see the antimicrobial mechanisms of Ag NPs described in Section 2.2) [74]. An example of the reinforcement of antimicrobial properties of polymers with biocidal Ag NPs was given by Song and coworkers, who successfully embedded Ag NPs in PMMA and in PTBAM nanofibers previously assembled via radical-mediated dispersion polymerization (Figure 10a,b) [190]. Both Ag/nanofiber-based composites exhibited great antimicrobial performance against *E. coli* and *S. aureus,* but, as compared to PMMA/Ag, the PTBAM/Ag nanocomposite exhibited better bactericidal attributes. Most probably, this was due to the antibacterial nature of the PTBAM substrate (in comparison, the PMMA substrate exhibits no antibacterial properties) [190]. The two bacteria mentioned above can be further targeted by a similar structure comprised of Ag NPs firmly grafted onto a self-assembled monolayer (SAM) of antimicrobial PEI on a glass substrate [193].

More recently, other antimicrobial nanocomposites containing Ag were developed. PCBMA/Ag and polylactide (PLA)/PEG/Ag nanocomposites are just two examples in which Ag was used to reinforce the antimicrobial properties of zwitterionic PCBMA [189] and the antiadhesive properties of PEG [114] polymers, respectively. In the first example, Li and coworkers have coordinated the Ag^+^ to the carbonyl groups located on PCBAM brushes, previously grafted on poly(vinylidene fluoride) (PVDF) membranes. Ag^+^ was then reduced to Ag NPs, leading to PVDF-*g*-PCBMA/Ag nanocomposites. Due to synergistic effects, the latter displayed improved hydrophilicity and antimicrobial properties than membranes made of only PCBMA brushes or pure Ag NPs [189]. In the second example, Turalija and coworkers have prepared antimicrobial films made of PLA and containing 5% of PEG (PLA/PEG). Employing a procedure of surface modification based on plasma technology, they have further incorporated Ag into PLA/PEG films, finally obtaining PLA/PEG/Ag film nanocomposites. By comparing the antimicrobial properties of these two types of nanocomposites against *S. aureus* and *E. coli*, it was found that plasma-deposited thin-films containing Ag exhibited enhanced hydrophilicity and better antimicrobial properties, most probably due to the good antimicrobial attributes provided additionally by Ag [114].

Besides Ag, Cu is also well-known for its antimicrobial properties [191], and thus, it can be used to reinforce the antimicrobial properties of polymer-based composites (in this work, we refer to examples where Cu is only used along with antimicrobial polymers, for other Cu-based antimicrobial systems, the readers are advised to look elsewhere [127,198,199,200]). Cu can kill bacteria by several mechanisms, including the release of Cu ions [201] or Cu NPs [202], as well as biofilm inhibition [203]. More details on these mechanisms can be consulted in the excellent review of Tamayo and coworkers [191]. Typical examples of the reinforcement of antimicrobial properties of polymers with biocidal Cu are depicted by the PEI/Cu NPs and pectin–Cu^2+^–PEI composites. The first example emphasizes an antimicrobial composite that was prepared by irreversibly binding positively charged Cu NPs, previously synthesized utilizing PEI as the capping agent, on the negatively charged surface of a polyamide-based membrane (Figure 10c) [188]. This composite was able, after 1 h of contact, to reduce about 80%, 87% and 96% of attached live *E. Coli*, *P. aeruginosa* and *S. aureus* bacteria, respectively (Figure 10d). The second example is depicting a Cu-containing polymer film composite prepared by thermal reduction of Cu^2+^ ions to metallic Cu in the pectin–Cu^2+^–PEI interpolyelectrolyte–metal complexes, i.e., by transferring electrons from the nitrogen atoms of amino groups of PEI to Cu^2+^ ions accompanied by the destruction of the metal-based complexes [115]. Resulting pectin-PEI-Cu heterogeneously structured composite exhibited better antimicrobial attributes against *S. aureus* and *E. coli* than did its counterpart pectin-PEI.

Other inorganic NPs with antimicrobial properties that can be employed to prepare antimicrobial polymer-based composites include Cu oxide, titanium dioxide, zinc oxide, Au, etc. Most of these NPs need to be incorporated into polymers due to their tendency to form aggregates, which significantly diminishes the antimicrobial potential. Moreover, polymeric chains can boost the germicidal effects by enabling the inclusion of additional antimicrobial moieties [204]. For instance, TiO_2_ compounds can disrupt bacterial cell membranes and kill bacteria by producing ROS in the presence of light. Coating TiO_2_ compounds with polymers leads to composites that are biocidal irrespective of light conditions [205]. Au NPs are considered to be weak bactericidal agents. However, recyclable antibacterial Au NP-polymer composites with targeted efficacy against pathogenic *E. coli* were manufactured [206]. Unfortunately, these composites are less potent against *S. aureus*, on which they cannot specifically attach due to the absence of sugar-binding fimbriae on the Gram-positive bacterial cells. Further relevant information on antimicrobial composites based on inorganic NPs can be found in the literature [204].

Antimicrobial polymers can be further combined with carbon-based nanomaterials. The latter include nanostructures, such as carbon nanotubes (CNTs) [195], graphene [207], graphene oxide (GO) [208], reduced graphene oxide (RGO) [209] and others, and are well-known for their applications in biomedical engineering [210] and other biological applications [211], including drug delivery [212,213], sequential enrichment of peptides [214], osteoporotic bone regeneration [215], enzyme immobilization [216], biomaterials and bionics [217], generation of neurons [218], cellular migration [219], etc. Most of these nanomaterials can be routinely produced [220,221] or synthesized [222,223,224], even in common organic solvents used to also dissolve polymers [209] and possess important antimicrobial properties [225,226,227,228,229,230]. Such materials kill microbes through their physical interaction with the microbial surface leading to localized degradation of microbial cell walls [231] through wrapping, insertion or nano-knife-like processes [232].

An example depicting a polymer-CNTs antimicrobial composite was given by Joo and coworkers, who have grafted various contents of poly(2-dimethylaminoethyl methacrylate) (PDMAEMA), a polymer known for its antimicrobial properties [233,234,235], onto bromine-functionalized multiwalled CNTs (MWCNTs) via an ATRP method [195]. The resulting PDMAEMA-MWNT composite (Figure 10e), optimized with respect to its content of PDMAEMA, exhibited clear antibacterial properties when tested against *S. aureus* and *E. Coli* [195]. Similarly, MWCNTs can be functionalized with antimicrobial amphiphilic poly(propyleneimine) (APPI) dendrimer to obtain reinforced antimicrobial MWCNTs-APPI composites that can efficiently kill *B. subtilis*, *S. aureus*, and *E. coli*. Bacteria killing efficiency can be further reinforced by adding Ag NPs to MWCNTs-APPI composite [196].

Besides CNTs, a low percentage of amine-terminated GO (GO–NH_2_) can also be used to reactively harmonize blends of low-density PE and PEG and to further develop porous antimicrobial membranes [116]. The resulting PE/PEG/GO–NH_2_ uniformly dispersed composite proved itself more efficient in destroying *E. coli* than its analog PE/PEG blend (demonstrated antibacterial efficiencies were 90% and 20%, respectively). More detailed information on nanocomposites based on antimicrobial polymers, as well as on recent strategies to kill microbes, can be further consulted in excellent publications available in the literature [74,97,191,232].

## 6. Conclusions

State-of-the-art medical devices, implants, wound dressings and contact lenses are just a few of the biomedical applications with significant impacts on human health and comfort. These applications require microbe-free surfaces that can be obtained inclusively via coating with polymers possessing antiadhesive and/or antimicrobial properties. A first advantage of the polymer-coating approach is the possibility to assemble polymer chains into well-defined structures and, thus, to tune the antiadhesive and antimicrobial properties of resulting surfaces. The choices are to design and synthesize polymers either with only antiadhesive (biopassive) or antimicrobial (bioactive) properties or with both microbes repelling and killing attributes. Here, experiments have proved that various polymeric structures (brushes, nanofibers, NPs, worms, vesicles, etc.) can reduce the adhesion of microbes on different surfaces from several to few tens of times and/or can totally kill them after the contact. A second advantage of utilizing polymers to generate sterile surfaces is the possibility to either blend polymers exhibiting antiadhesive and/or antimicrobial properties to obtain new structures with enhanced antimicrobial attributes or to exploit the synergistic antimicrobial effects within nanocomposites made of microbe repelling/killing polymers and other materials displaying biocidal attributes (inorganic NPs, carbon-based materials, etc.). In these cases, blends and nanocomposites can exhibit either bifunctional (i.e., bioactive and biopassive) antimicrobial mechanisms or can simply reinforce the bioactive antimicrobial mechanisms. Both situations lead to polymer-based systems with enhanced capability to prevent and suppress the growth of various undesired microorganisms, including bacteria. Moreover, these systems exhibit antimicrobial mechanisms that cannot be outwitted by pathogens and thus, can combat, for example, the bacterial resistance to antibiotics. Consequently, polymer-based antimicrobial systems could become a viable alternative to antibiotics and disinfectants, especially if the research efforts in this direction further intensify. More exactly, significant improvements could be made by developing novel and more efficient biocidal moieties, by designing and optimizing antimicrobial polymer chains with the ability to form unique ordered structures and to attach to well-defined surfaces, or by synthesizing carbon-based materials in organic solvents with the aim to boost the preparation of the next-generation of antimicrobial polymer-based nanocomposites.

## Figures and Tables

**Table 1:** Summary of the main antimicrobial structures used to develop various mechanisms to repel and destroy microbes.

Antimicrobial Polymer Devices	Employed Polymeric Structures	Antimicrobial Principle	Ref.
Drug-loaded polymers	Nanoparticles, micelles, vesicles, dendritic structures	Delivery and release of drugs or other biocidal components	[30,31,32,33,34]
Polymeric hydrogels	Gel-like microstructures	Employment of drugs/biocides to kill microbes	[35,36]
Surface-bound polymers	Various structures: (bottle)brushes, spherical nanoparticles (micelles, vesicles), rods, fibers, worms, multilayers, etc.	Neutral polymer-based surfaces (steric repulsion) Anionic polymer-based surfaces (electrostatic repulsion) Ultrahydrophobic (low-energy) polymer-based surfaces Contact killing surfaces (cationic, use of biocidal moieties) Biocide releasing surfaces (use of biocides) Stimuli-responsive surfaces (temperature, pH, etc.) Adaptive bactericidal surfaces	[37,38,39,40]

**Table 3:** Summary of polymeric systems and configurations that can be employed to kill various microbes on surfaces. *Log* denotes log10 of colony-forming units CFU/mL and refers to the bacteria removal value defined as the logarithm ratio of the bacterial concentration measured at a specific time with respect to the initial bacterial concentration. Efficacy in *%* refers to the bacteria kill ratio.

Antimicrobial Polymer	Configuration/ Nanostructure	Dimension	Antimicrobial Mechanism	Efficacy	Microbe of Interest	Ref.
PLys-*b*-PHPMA	Spheres, worms, vesicles	50–200 nm diameter	Bioactive	3.4 *log*	*S. epidermidis*	[150]
P(DMA-*co*-APMA)	Brushes	~8–42 nm thick	Bioactive	-	*P. aeruginosa*	[143]
Polypeptides	α-Helical structure	~0.232 nm radius	Bioactive	~100%	*E. coli*, *S. aureus*	[149]
P(DMA-MEA-DQA)	Oriented thin-films with catechols	-	Bioactive	100% 85%	*E. coli* *S. aureus*	[109]
P(DMEMA-*co*-MMA)/PEGDMA	SIPN	800 nm thick	Bioactive	5 *log* 5 *log*	*S. epidermidis* *E. coli*	[152]
PDDA/PMMA	Core-shell NPs	A few hundred nm diameter (in aqueous medium)	Bioactive	8 *log* 7 *log* 2 *log*	*E. coli* *S. aureus* *C. albicans*	[153]
PDDA/PMMA	Core-shell NPs	94 nm thick	Bioactive	7 *log*	*E. coli*, *S. aureus*	[154]
PEI-PEOX	Thin-films	77 nm thick	Bioactive	*>98%*	*S. aureus, E. coli*	[110]
N-halamine PCHP, PAHP	Multilayered films	-	Bioactive	*100%* *99.73%*	*S. aureus* *E. coli*	[111]

**Table 4:** Summary of polymeric systems and configurations that exhibit both antiadhesive and antimicrobial properties on surfaces. Following abbreviations were used: *Fusarium (F.)*, *Bacillus (B.)* and *species* (sp.). Efficacy in % refers to the bacteria kill ratio or bacterial adhesion reduction.

Antimicrobial Polymer	Configuration/ Nanostructure	Dimension	Antimicrobial Mechanism	Efficacy	Microbe of Interest	Ref.
APEG_2400_–PHMB	Bottlebrushes	25 nm thick	Biopassive + bioactive	5 *log*	*E. coli*	[170]
APEG_2400_–PHMG	Bottlebrushes	20 nm thick	Biopassive + bioactive	*>99.9%*	*P. aeruginosa, S. aureus, F. solani*	[171]
PF-127	Binary brushes	~7–14 nm thick	Biopassive + bioactive	*85%*	*B. subtilis*	[169]
PEI/SMA/PEI	Pores in thin films	~100 nm diameter	Biopassive + bioactive	*99.99%*	*E. coli*	[176]
PEGMA/PMETA	Multilayered films	10–14 nm bilayer	Biopassive + bioactive	*97%*	*Pseudomonas* sp.	[112]
PEG-polypeptides	Bottlebrushes	1–2 µm thick	Biopassive + bioactive	*>99%*	*E. coli*, *S. aureus*, *P. aeruginosa*	[174]
PMPC/a-PMETA	Assembled binary brushes	59 nm thick	Biopassive + bioactive	*93%* *93%*	*S. aureus* *A. coffeaeformis*	[177]
MePPEP/ MePSAR	Assembled binary brushes	310 nm thick	Biopassive + bioactive	*99%/97%* *99%/99%* *99%/94%* *99%/95%*	*S. aureus* *E. coli* *P. aeruginosa* *C. albicans*	[178]
PEG-*b*-PC	Brushes	5–7 nm thick	Biopassive + bioactive	*100%*	*S. aureus*	[173]

**Table 6:** Summary of the polymer-based nanocomposites that can be efficiently employed against various microorganisms on surfaces.

Blend/Composite	Configuration/ Nanostructure	Dimension	Antimicrobial Mechanism	Efficacy	Microbe of Interest	Ref.
PMMA/Ag, PTBAM/Ag	Nanofibers	40 nm diameter, 10 μm long	Bioactive reinforced	*-*	*E. coli, S. aureus*	[190]
PEI/Ag	NPs grafted on SAM	10–14 nm thick (total)	Bioactive reinforced	*~6 log* *0.86 log*	*E. coli* *S. aureus*	[193]
PVDF-*g*-PCBMA/Ag	Pores/brushes	-	Bioactive reinforced	*-*	*E. coli*, *S. aureus*	[189]
PLA/PEG, PLA/PEG/Ag	Films; NPs	~40 μm thick 25 nm thick	Biopassive + bioactive	*-*	*E. coli*, *S. aureus*	[114]
P2VP-*b*-PEG	Smart micelles	60 nm (unloaded)	Bioactive		*-*	[194]
PEI/Cu	Positively charged NPs	34 nm radius	Bioactive reinforced	87% 96% 80%	*E. coli* *P. aeruginosa* *S. aureus*	[188]
Pectin–PEI–Cu	Films with Cu NPs	100 μm thick	Bioactive reinforced	-	*S. aureus*, *E. coli*	[115]
PDMEMA-MWCNTs	Nanotubes	26 nm diameter	Bioactive reinforced	42% -	*E. coli* *S. aureus*	[195]
MWCNTs-APPI/MWCNTs-APPI–Ag NPs	Nanotubes Ag NPs	15 nm diameter 15 nm diameter	Bioactive reinforced	96%/99% 96%/99% 87%/93%	*B. subtilis* *S. aureus* *E. coli*	[196]
PE/PEG/GO–NH_2_	Films	-	Bioactive reinforced	90%	*E. coli*	[116]
